# Chemotherapy induced microsatellite instability and loss of heterozygosity in chromosomes 2, 5, 10, and 17 in solid tumor patients

**DOI:** 10.1186/s12935-014-0118-4

**Published:** 2014-11-30

**Authors:** Nasir Kamat, Mohammed A Khidhir, Sabir Hussain, Mouied M Alashari, Ulf Rannug

**Affiliations:** Department of Molecular Biosciences, the Wenner-Gren Institute (MBW), Stockholm University, SE-106 91 Stockholm, Sweden; Department of Genetics Research, Management of Natural Conservations, AlAin City, UAE; Department of Oncology and Hematology, Tawam Hospital, AlAin City, UAE; Department of Pathology, University of Utah, Salt Lake City, Utah 84112 USA

**Keywords:** Chemotherapy, Genetic instability, Microsatellites, Mismatch repair, Secondary tumors

## Abstract

**Background:**

The inevitable side effects of the currently used chemotherapy are associated with serious syndromes. Genotoxic effects and consequent genetic instability may play an important role in these syndromes. The aim of the study was to evaluate chemotherapy-related microsatellite instability (MSI), loss of heterozygosity (LOH), and loss of mismatch repair (MMR) expression in solid tumor patients.

**Methods:**

Samples were collected from 117 *de novo* patients with solid tumors of different origins. Specimens, taken pre- and post-treatment, were screened for MSI and LOH in 10 microsatellite sequences in blood, and expression of five MMR proteins were analyzed in cancer tissues using immunohistochemistry. Statistical analysis included the use of; Fisher’s exact test, Chi Square, and an inter-rater reliability test using Cohen’s kappa coefficient.

**Results:**

Microsatellite analysis showed that 66.7% of the patients had MSI, including 23.1% high-positive MSI and 43.6% low-positive MSI. A large portion (41%) of the patients exhibited LOH in addition to MSI. MSI and LOH were detected in seven loci in which incidence rates ranged from 3.8% positive for Bat-26 to 34.6% positive for Tp53-Alu. Immunohistochemistry revealed that human mutL homolog 1 (hMLH1) expression was deficient in 29.1% of the patients, whereas 18.8%, 23.9%, 13.4%, and 9.7% were deficient for human mutS homolog 2 (hMSH2), P53, human mutS homolog 6 (hMSH6) and human post-meiotic segregation increased 2 (hPMS2), respectively. There was a significant correlation between MSI and LOH incidence in Tp53-Alu, Mfd41, and APC with low or deficient expression of hMLH1, hMSH2, and P53. A significant association between MSI and LOH, and incidence of secondary tumors was also evident.

**Conclusions:**

The negative correlation between MMR expression, MSI, and LOH and increased resistance to anti-cancer drugs and development of secondary cancers demonstrates a useful aid in early detection of potential chemotherapy-related side-effects. The diagnostic value demonstrated in our earlier study on breast cancer patients was confirmed for other solid tumors.

**Electronic supplementary material:**

The online version of this article (doi:10.1186/s12935-014-0118-4) contains supplementary material, which is available to authorized users.

## Background

Genomic alterations in patients receiving chemotherapy, especially alkylating agents, lead to many abnormal clinical phenotypes. Increased resistance to chemotherapy treatments and generation of secondary cancers, e.g. secondary acute myeloid leukemia (AML) and/or myelodysplasia have been reported with incidence rates of 1-5% [1-4]. Chemotherapy-related AML was described in the 1980s [5,6], followed by a wider range of investigations including all types of cytotoxic agents. In many cancer patients, AML has been detected from a few months to several years after chemotherapy treatment [2,3,5,7-10]. Genetic alterations that may be the cause of secondary neoplasms include both germline and somatic mutations, and one or more mechanisms (point mutations, deletions, mitotic recombination, gene conversion, non-disjunction and chromosomal loss, or rearrangement of genes) can be implicated [11-13].

Simple tandem repetitive DNA sequences consisting of arrays of one to five base pairs (i.e., microsatellites) have been used as molecular biomarkers in kinship, population genetics, linkage mapping, and other studies [14-17]. These DNA sequences are particularly prone to mutations generating new allele lengths via insertion–deletion loop formation during DNA synthesis [18,19]. Microsatellites can also serve in studying lesions at the gene level, such as duplication or deletion [20,21]. Microsatellite instability (MSI) and loss of heterozygosity (LOH) are frequently described abnormalities known to be some of the early steps in the tumorigenesis pathway [22-24], and correction of MSI and LOH requires sufficient expression and activity of MMR proteins [25]. Genetic alterations are normally corrected by the mismatch repair system (MMR), which employs several proteins including human mutL homolog 1 (hMLH1), human mutS homolog 2, 3 and 6 (hMSH2, hMSH3, and hMSH6, respectively), and human postmeiotic segregation increased1 and 2 (hPMS1 and hPMS2) that are known to interact with P53 protein and tumor suppressor genes [26-28]. Interaction between MMR and tumor suppressor genes is an integral part of repairing damages in microsatellite sequences and reducing potential carcinogenesis in affected patients [29,30]. However, when affected alleles are within tumor suppressor genes, this may lead to loss of function of an active tumor suppressor protein [31,32], which indicates the necessity to assess the genetic alterations within that gene. Moreover, a study conducted in a murine model has demonstrated the trans-generational nature of genotoxic effects of anti-cancer drugs. The genetic alterations appeared with an increased frequency in germline and somatic cells of the first-generation offspring of the treated animals [33,34].This trans-generational effect naturally raises concerns about similar genetic instability in children of anticancer therapy survivors.

Treatment-related MSI and LOH have been analyzed in a few studies in recent years, highlighting the importance of screening patients for these genomic instabilities after chemotherapy completion [35-37]. Consequences of MSI and LOH that were observed in these studies included increased resistance to chemotherapy, recurrence of primary tumors, and/or appearance of secondary malignancies. However, these studies were exclusively conducted on breast cancer patients. Investigating the possibility of similar outcomes in patients with other cancers is necessary to determine if this is a more widespread phenomenon.

Following the results reported in our previous study on breast cancer patients [37], a new study was similarly designed to assess whether patients with other solid tumors are predisposed to higher genetic instability linked to chemotherapy treatment. In addition, the present study includes an extended follow-up period of 52 months to verify if it is possible to correlate MSI, LOH, and reduced MMR expression with clinical findings. We believe that such results would point to the existence of a link between chemotherapy-related genetic instability and clinical phenotypes of more chemo-resistant cancers or appearance of secondary tumors.

## Results

### Blood samples

**Figure 1:**
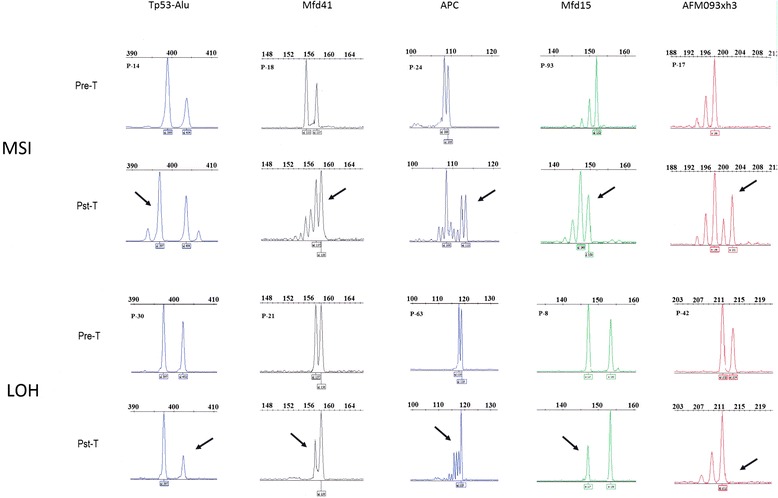
**Typical microsatellite instability (MSI) and loss of heterozygosity (LOH) in five microsatellites in post-treatment samples from several patients.** P## represent the patient’s number. Pre-T = pre-treatment specimen analysis, Pst-T = post-treatment analysis. Arrows point to the lesion in post-treatment analyses. In two of the LOH cases also new alleles, representing simultaneous MSI, could be seen. Different dyes were used to label overlapping markers (detailed markers characteristics including used dyes are displayed in Additional file 3: Table S2.

Screening of 10 microsatellite markers revealed that 78 of 117 solid tumor patients (66.7%) had microsatellite instability in at least one locus. Based on National Cancer Institute (NCI) criteria, 27 patients (23.1%) were found to be high-positive MSI (MSI-H) due to MSI and LOH in two or more microsatellites sequences, and 51 patients (43.6%) exhibited single MSI events and were classified as low-positive MSI (MSI-L). LOH was detected in 48 patients (41%). No MSI or LOH was detected in any of the tested markers in 39 patients (33.3%); this group was classified as microsatellite stable. No MSI or LOH events were detected in the pre-treatment specimens (Table 1). The total incidence of MSI in the blood samples collected was 108/351 (30.7%). However, the incidence decreased with time. MSI detected in post-treatment specimens was reduced from 78 (66.6%) in the first to 30 (25.4%) in the second post-treatment samples, and this is compatible with the transient nature of MSI lesions. The number of LOH events, 48 giving a total of 27.4%, was evenly distributed between the two post-treatment groups (Table 1). Detailed individual incidences of MSI and LOH (Table 2) showed that there was a significant occurrence of these instabilities in five of the seven markers. Tp53-Alu on chromosome 17 was the marker most susceptible to error; 34.6% of the positive patients exhibited mutations in this locus. Less frequent but significant incidences of MSI/LOH were observed in Mfd41and Mfd15 on chromosome 17, APC on chromosome 5, and AFM093xh3 on chromosome 2 (26.9%, 19.2%, 21.8%, and 14.5%, respectively, Table 2). Alleles 399 and 402 of Tp53-Alu, allele 157 of Mfd41, allele 198 of AFM093xh3, alleles 147 and 152 of Mfd15, and allele 109 of APC all demonstrated instability and LOH more frequently than other alleles tested (Figure 1). MSI appeared in Tp53-Alu as deletions or insertions of one to a few base pairs, these events occurred with similar frequencies, indicating no inherent preference for one event over the other. LOH formed the majority of events at this locus, and it most often resulted in nearly 100% decrease in allele signal. Changes in Mfd41 included shortening of the original allele peak height and the addition of new peaks on both sides of the allele with a higher tendency of deletions (i.e., shorter sequences). LOH in alleles 109 and 118 of APC was accompanied by the emergence of a novel allele of 162 nucleotides (Additional file 1: Figure S1). A reference group of healthy individuals was screened using the same microsatellites panel. The results showed transient MSI-L in two individuals; one in Mfd15 and the other in TP53.PCR15 loci (Table 1). Both transient MSI-L events disappeared in the second samples from the healthy individuals. No LOH events were detected in the blood samples from the reference group.

**Table 1:** **Incidences of MSI-L, MSI-H, and LOH in blood samples in patients and reference groups from the three sampling events**

**Group**	**No. of samples**	**No. of MSI- L**	**No. of MSI-H**	**No. of LOH**
**Patients**				
Pre-treatment	117	0	0	0
Post-treatment 1	117	51	27	48
Post-treatment 2	117	30	0	48
**Reference (healthy)**				
Pre-treatment	60	0	0	0
Post-treatment 1	60	2	0	0
Post-treatment 2	60	0	0	0

LOH at alleles 109 and 118 of APC was accompanied by the appearance of a novel allele of 162 base pairs (see Additional file 1: Figure S1). Furthermore, the incidence rate versus age correlation showed that the incidence of MSI/LOH increased with age (see Additional file 2: Figure S2). Cancer-specific incidences of instability showed that pancreatic, lung, and gastric cancer patients had high incidences of MSI (Table 4).

**Table 2:** **Incidence rate of MSI and LOH, number of alleles isolated, and allelic imbalance noticed for each marker**

**Marker**	**Chr./Locus**	**No of positive patients**	**% of positive MSI**	**No. of alleles isolated**	**Allelic imbalance**
Mfd15	17q11.2	15	19.2%	7	L 0–0.69 U 1.56-2.72
APC	5q21/22	17	21.8%	8	L 0–0.69 U 1.5-2.61
Tp53-Alu	17p13.1	27	34.6%	12	L 0–0.67 U 1.48-2.53
Mfd41	17p12-11.1	21	26.9%	8	L 0–0.59 U 1.35-2.76
Bat-25	4q12	0	0%	4	L 0.71-0.77 U 0.94-1.2
TP53.PCR15	17p13.1	0	0%	5	L 0.80-0.86 U 0.92-1.23
AFM093xh3	2p16	11	14.1%	6	L 0–0.68 U 1.51-2.44
Bat-40	1p13.1	0	0%	4	L 0.73-0.81 U 0.91-1.31
Bat-26	2p	3	3.8%	4	L 0.23-0.62 U 1.31-1.65
Mfd28	10pter	7	8.9%	5	L 0.2-0.64 U 1.37-1.95

L = lower allelic imbalance. U = upper allelic imbalance range.

### Cancer tissues

Immunohistochemistry demonstrated that chemotherapy treatment induced a loss of expression of MMR proteins. In pre-treatment specimens, hMLH1was deficient in 22 patients (22%), whereas in post-treatment specimens hMLH1 found to be deficient in 51 patients (51%), which indicate a loss of expression of this MMR protein in 29% of cancer tissue (p <0.0001) after receiving chemotherapy. Furthermore, the pre-treatment specimens showed that hMSH2, P53, hMSH6, and hPMS2 were deficient in 16, 27, 11, and 9 patients, whereas post-treatment specimens were deficient in 35, 51, 25, and 19 patients, respectively. These deficiencies accounted for 18.8% (p <0.0001), 23.9% (p <0.0001), 13.4% (p = 0.0001), and 10.2% (p <0.0001) in hMSH2, P53, hMSH6 and hPMS2, respectively.

Statistical analysis showed a significant correlation between low or deficient expression of hMSH2 and MSI/LOH in Tp53-Alu, Mfd41, and APC (Table 3). Low P53 expression significantly correlated with MSI and LOH in Tp53-Alu and APC, whereas low or deficient expression of hMLH1 correlated with MSI and LOH in only Tp53-Alu (Table 3). Clinical follow-up studies for up to 52 months revealed that 19 patients (16.2%) were diagnosed with clinical complications of increased resistance to the applied chemotherapy; 15 patients (12.8%) had a recurrence of primary tumors and 4 patients (3.4%) developed secondary tumors (Table 4). All 19 complicated cases were patients previously diagnosed to have chemotherapy-related MSI and LOH after the completion of their treatment regimen (13 patients [11.1%] with MSI-H and 6 [5.1%] with MSI-L). Fisher’s exact test indicated a significant association between MSI/LOH and the incidence of secondary tumors (two-sided Fisher’s exact p = 0.018, and one-sided Fisher’s exact p = 0.012).

**Table 3:** **Correlation of MSI/LOH incidence to the low expression of MMR genes**

		**Mfd41**	**Tp53-alu**	**Mfd15**	**APC**	**AFM093xh3**
hMLH1	Kappa	−0.111	0.198	−0.167	−0.046	−0.153
	p-value	0.212	0.031	0.041	0.587	0.078
	Significance Level	Ns	**	Ns	Ns	Ns
hMSH2	Kappa	0.761	0.776	0.011	0.479	0.011
	p-value	< 0.0001	< 0.0001	0.899	< 0.0001	0.312
	Significance level	***	***	Ns	***	Ns
P53	Kappa	0.139	0.559	−0.145	0.213	−0.125
	p-value	0.129	< 0.0001	0.093	0.016	0.088
	Significance level	Ns	***	Ns	**	Ns
hMSH6	Kappa	0.023	0.101	−0.115	0.033	−0.178
	p-value	0.503	0.413	0.121	0.311	0.052
	Significance level	Ns	Ns	Ns	Ns	Ns
hPMS2	Kappa	0.142	−0.206	−0.162	0.086	−0.107
	p-value	0.119	0.312	0.241	0.311	0.189
	Significance level	Ns	Ns	Ns	Ns	Ns

**refers to moderately significant correlation.

***refers to highly significant correlation.

**Table 4:** **Cancer types specific results (ratio to the cohort, low and high-positive MSI in each type)**

**Cancer type**	**No. of cases**	**%: study cohort**	**MSI- L**	**MSI-H**	**Recurrence**	**Secondary CAs**
Gastric CA	20	17.1%	11	6	4	1
Nasopharyngeal CA	19	16.2%	6	4	2	1
Ovary CA	13	11.1%	5	3	2	0
Lung CA	12	10.3%	7	3	3	1
Pancreas CA	9	7.7%	5	3	2	1
Squamous carcinoma	9	7.7%	5	1	0	0
Prostate CA	9	7.7%	3	0	0	0
Anal canal CA	6	5.1%	1	2	0	0
Germ cell tumor	6	5.1%	2	2	1	0
Oral cavity	4	3.4%	2	0	0	0
Uterus sarcoma	4	3.4%	2	0	0	0
Urinary bladder CA	3	2.6%	1	2	0	0
Glioblastoma CA	2	1.7%	1	0	0	0
Ewing sarcoma	1	0.9%	0	1	1	0

## Discussion

The occurrence of chemotherapy-related genetic instability, especially MSI and LOH, is documented in several studies [38-40]. The importance of studying these instabilities is emphasized by the role MSI and LOH play in the early stages of tumorigenesis and cancer development [22-24]. The assessment of MSI and LOH may carry a predictive value for earlier detection and management of secondary tumors [37,41]. Since previous reports indicate that the use of 10 markers is optimal, the present study used a panel of five microsatellites recommended by the NCI guidelines and another five markers of our choosing [42-44]. The results reported so far, including the present study, show varied incidences of MSI and LOH in many types of cancers [45-47]. Our investigation revealed MSI and LOH mutations to occur in 7/10 markers studied, a total MSI incidence of 66.7% among mixed origin cancer patients represents a significant level of chemotherapy related genetic instability.

MSI and LOH events appeared to be most prevalent in the Tp53-Alu marker, although Mfd41 and APC also exhibited significant incidences of MSI and LOH (Table 2). These results do not seem to be due to spontaneous occurrence, given that the individuals in the reference group did not show any changes in these microsatellite sequences. In addition, MSI and LOH events significantly correlated with low expression of MMR proteins (Table 3). These findings support the hypothesis that malfunctions of the MMR, along with the error-prone nature of microsatellite sequences, contributes to the development of MSI and LOH. Previous studies report that chemotherapy-induced MSI and LOH triggers secondary AML and myelodysplastic syndrome in 1-2% of cancer patients within a few months to several years after completion of chemotherapy [1-3,7]. Recent studies with larger cohorts and broader selection of tumor types suggest an even higher incidence (up to 5%) of secondary tumors [4]. Additionally, secondary AML is believed to occur in 10-30% of all diagnosed cases of AML [3]. The elevated incidence of secondary AML in more recent studies could be due to longer survival times for chemotherapy treated patients, and the administration of new generations of DNA-damaging chemotherapies (particularly alkylating agents and platinum-based anti-cancer drugs). It is not yet fully understood if somatic mutations predispose carriers to specific tumors, or at minimum, mediate the development of resistant phenotypes of cancer.

In reviewing the incidence of MSI and LOH in different solid tumors (Table 4), we observed that gastric, lung, ovarian, nasopharyngeal, pancreatic, squamous cell carcinoma, and prostate cancers (which together form 78% of our cohort) showed varied incidence of MSI. Incidence of MSI ranged from 33.3% in prostate to 83-87% in lung, pancreatic and gastric cancers, and showed a strong correlation with deficiencies of MMR proteins. Follow-up studies for up to 52 months showed that undesirable effects of chemotherapy were detected in those patients who previously were found to harbor MSI and LOH mutations and show MMR deficiency. These effects are presented clinically as resistance to chemotherapy regimen and recurrence of the primary disease in 15 patients (12.8%), or developing secondary cancers or myelodysplastic syndrome in 4 patients (3.4%; Table 4). Previous studies have shown that although immunohistochemistry has an advantage over MSI analysis in identifying MMR deficient samples, it is incapable of distinguishing MMR proteins with impaired function from normally functioning proteins [48,49]. The four major MMR proteins (hMLH1, hMSH2, hMSH6, and hPMS2) are not equally engaged in the repair of different length microsatellite instability. hMSH6 is involved primarily in the repair of single base-base mismatches or single base deletions/insertions, but appears to have no functional role in the repair of dinucleotides or longer mismatches [49]. This variety in MMR protein roles highlights the necessity of combining microsatellite instability analysis with MMR proteins immunohistochemistry in screening cancer patients.

LOH in tumor suppressor inhibitor of growth 1 (*ING1*) is associated with carcinogenesis in human non-small cell lung cancer [50], and deletion in the neurofibromin 1 (*NF1*) gene promotes formation of malignant melanoma [51]. Our results add to this evidence by demonstrating somatic mutations leading to LOH in another tumor suppressor gene (*Tp53*-alu, chr. 17p13.1). Another important finding of this study was the MSI and LOH observed in *APC*; LOH in certain alleles of APC was accompanied with the emergence of a novel allele at this locus. Whereas APC plays a role in hereditary nonpolyposis colorectal cancer (HNPCC) [52], and is methylated in the tumorigenesis of other cancers [53,54], mutated APC may be of value in predicting secondary malignancies if more data are made available about its status during tumorigenesis of secondary cancers.

A murine study designed to assess the profundity and persistence of the genotoxic effects of chemotherapy drugs in current use showed that chemotherapy drugs pose a trans-generational risk manifested in higher mutation rates and genetic instability in the first generation of the treated animals [33,34]. These results may justify concerns about similar genetic instabilities in children of anticancer chemotherapy surviving patients.

Insertion/deletion mutations and the error-prone nature of microsatellites are often implicated in the development of chemotherapy-related secondary malignancies. Recent findings suggest an involvement of myeloid/lymphoid or mixed-lineage leukemia rearrangement events as a triggering mechanism that mediates the development of secondary AML after completion of chemotherapy treatment using Etoposide [11], or a combination of Methotrexate, Cisplatin, doxorubicin, and Etoposide [7].

A predictive value for MSI and LOH in HNPCC was determined after use in molecular profiling and survival studies in such patients [41,55]. Recent findings support the role of somatic mutations in tumor suppressors in cancers developing resistance to chemotherapies [56,57]. This may be explained by emphasizing the mode of action of many chemotherapies; these treatments aim to trigger programmed cell death in cancer cells, a process largely mediated by tumor suppressors like the tumor protein 53 (*Tp53*) and retinoblastoma (*RB*) genes. Analysis of MSI and LOH in sequences linked to these genes is likely to be of predictive value in tumorigenesis that is known to occur through inactivation of these genes. Although our cohort was predominantly younger (68 patients less than 51 years of age, see Additional file 2: Figure S2), our results indicate that MSI and LOH increased with age, likely due to the synergistic effect between chemotherapy-induced genetic instability and age-related increased cellular stress.

MSI and LOH play an important role in the early stages of tumorigenesis. The frequency with which MSI and LOH present as adverse effects of currently used chemotherapy drugs, along with the important role LOH plays in inactivating tumor suppressor genes, demonstrates the importance of MSI and LOH as indicators and valuable bio-markers in screening cancer patients after completion of their chemotherapy regimen. Moreover, screening of microsatellite sequences that are directly related to tumor suppressors or are physically adjacent to these genes may aid in the earlier prediction of secondary neoplasm and is likely to provide a better understanding of tumorigenesis, which in turn may lead to more efficient alternative treatments.

## Methods

### Study design and chemotherapy regimen

The inclusion rule applied in this study was that only patients receiving chemotherapy for the first time were selected. Patients with colon or breast cancers and those with family history or evidence of higher tendency to develop tumors were excluded. Peripheral blood was collected for genetic analysis and tumor tissues that were biopsied or resected from patients were retrieved from pathology departments to perform immunohistochemistry testing for the expression of MMR proteins. Follow-up studies were performed for 48–52 months after completion of the chemotherapy regimen to monitor the presence of clinical complications (especially resistance to chemotherapy, recurrence of primary disease, and/or development of secondary cancers).

Patients were monitored for chemotherapy-related MSI and LOH, MMR expression, and tumor recurrences or development of secondary tumors. Chemotherapy regimens administered in the management of the tumors that were sampled in this study are displayed in Additional file 3: Table S2. Our research conformed to the Helsinki declaration and local legislations, and has been approved by the AlAin Medical District Human Research Ethics Committee at the Faculty of Medicine and Health Sciences, University of United Arab Emirates, under ethical permit no. AAMD/HREC 08/15. All participating patients signed consent forms after receiving a verbal and written information sheet.

### Blood samples and cancer tissues collection

A total of 351 peripheral blood samples were collected from 117 *de novo* cancer patients with a mean age of 54 years (18–89 years). Sampled tumors included: 20 gastric carcinoma (CA), 19 nasopharyngeal cancer, 13 ovarian cancer, 12 lung cancer, 9 pancreatic CA, 9 prostate CA, 9 squamous cell CA, 6 anal canal cancer, 6 germ cell tumors, 4 oral cavity cancer, 4 uterus sarcoma, 3 urinary bladder CA, 2 glioblastoma, and 1 case of Ewing’s sarcoma. Sampling was conducted on three occasions starting at 4–5 weeks prior to the first chemotherapy session (pre-treatment sample) to serve as a baseline, and two consecutive draws at 12-week intervals after the first collection. Initial post-treatment samples (post-treatment-1) were used to investigate the presence of MSI and LOH, and second post-treatment samples (post-treatment-2) were used to determine the persistence of the initial findings. One hundred eighty blood samples were collected from 60 healthy individuals who had never reported symptoms relevant to any neoplastic case. Sampling of the reference group followed the same protocol applied to patients. Samples of 196 cancer tissues resected from patients were collected from the pathology department for MMR protein expression analysis using immunohistochemistry.

### DNA extraction and LOH and MSI analysis

Genomic DNA was extracted from whole blood using 200-μl blood DNA kits, on a Biorobot EZ1 workstation (Qiagen Inc, Valencia, California, USA,). DNA yield was quantified using absorbance at A_260_ on a Beckman Coulter DU 650 spectrophotometer with special applications for nucleic acid quantification (Beckman Coulter Inc, Brea, California, USA). DNA purity, checked on A_260_/A_280_ absorbance, averaged 1.72.

Single and multiplex polymerase chain reactions (PCR) were conducted to amplify 10 loci (Bat-40, AFM093xh3, Bat-25, APC, Mfd15, Mfd41, Mfd28, Bat-26, Tp53-Alu and TP53.PCR15) on chromosomes 1, 2, 4, 5, 10, and 17. Fluorescently labeled primers were used to amplify the selected loci as previously described [58], and are detailed in Additional file 3: Table S2. Amplification reactions were conducted in 10 μl reaction volume of 1× Gold Amplitaq Master Mix (Applied Biosystems, Carlsbad, California, USA) with the addition of 80 ng purified genomic DNA, and adjusted to a final primer concentration of 0.4 μM. Cycling conditions were as follows: initial denaturation at 95°C for 5 minutes; 29 cycles of 94°C for 45 sec, 50–62°C for 30 sec, and 72°C for 50 seconds; and a final 50 minute extension at 70°C. PCR products were denatured in Hi-Di formamide, pooled with LIZ 500 GS internal molecular weight control and loaded on an ABI 3130 genetic analyzer (Applied Biosystems, Carlsbad, California, USA). Fragments were measured and compared using Gene-Mapper software Version 4 (Applied Biosystems, Carlsbad, California, USA). GeneScan data were obtained using a minimum peak detection limit of 50 relative fluorescent units and applying the local Southern size calling method. MSI irregularities were demonstrated by comparisons between the numbers and allele arrangements of pre-treatment and post-treatment samples. Specifically, new peak addition or the presence of novel alleles indicated MSI [59], whereas LOH was indicated when the peak height of one of the two heterozygote alleles was reduced by at least 35% [60,61]. GeneMapper Software Version 4 was used to calculate LOH.

### MMR analyses

Archived tissue samples were available from 101 patients. In three cases, the available tissues were not sufficient for the procedure. Two specimens from each patient were tested for MMR protein expression (pre and post-treatment). The 196 cancer tissues were embedded in paraffin blocks for MMR protein expression analysis. The TP-125 HLX Ultravision Plus Anti-Polyvalent HRP detection system (Lab Vision, Fremont, California, USA) using specific monoclonal antibodies for hMLH1, hMSH2, hMSH6, hPMS2 and P53 (Cell Marque, Rocklin, California , USA, California) was used in the staining procedure. Results were marked as positive when 10% or more of cells stained positively (nuclear stain). Healthy tissue from each patient and the standard controls supplied by the manufacturer were used as internal controls.

### Statistical analyses

Confidence intervals were calculated at the 95% and 99% levels. Fisher’s exact test and Chi Square were used for statistical analyses with the SPSS statistical analysis package. Additionally, an inter-rater reliability test using Cohen’s kappa coefficient was used to measure correlation between the MSI and LOH results and low MMR protein expression [62].

## Additional files

Additional file 1: Figure S1. Emergence of a novel allele of 162 nucleotides following the loss of heterozygosity in alleles 109 and 118 of APC. Additional file 2: Figure S2. Positive correlation between rates of MSI and LOH and the patient’s age. As patient’s age increases, the incidence of MSI and LOH appears to increase. Additional file 3: Table S1. Frequently used chemotherapy treatments in managing the sampled solid tumors in the current study. **Table S2.** The specific characteristics of the analyzed microsatellite markers.

## Abbreviations

AML: Acute myeloid leukemia
HNPCC: Hereditary nonpolyposis colorectal carcinoma
hMLH1: Human mutL homolog 1
hMSH2: Human mutS homolog 2
hMSH6: Human mutS homolog 6
hPMS1: Human post-meiotic segregation increased 1
hPMS2: Human post-meiotic segregation increased 2
LOH: Loss of heterozygosity
MMR: Mismatch repair
MSI: Microsatellite instability
NCI: National Cancer Institute
NER: Nucleotide excision repair
NIN: Nucleotide instability
PCR: Polymerase chain reaction
p53: p53 tumor suppressor protein
