# Supplementary material for: Chemotherapy induced microsatellite instability and loss of heterozygosity in chromosomes 2, 5, 10, and 17 in solid tumor patients
Source: Cancer Cell Int. 2014 Nov 30;14:118. doi: 10.1186/s12935-014-0118-4 (PMC4260186; doi:10.1186/s12935-014-0118-4)
Supplement: Additional file 1: Figure S1. — Emergence of a novel allele of 162 nucleotides following the loss of heterozygosity in alleles 109 and 118 of APC. [file 12935_2014_118_MOESM1_ESM.tiff]

Supplement Table 1. Frequently used chemotherapy treatments in managing the sampled solid tumors in the current study.

Cancer type	Chemotherapy regimen	Reference
**Gastric CA**	Epirubicin 50 mg/m^2 iv bolus d1 q3w x 8 cycles<br>Cisplatin (CDDP) 60 mg/m^2 iv d1 q3w x 8 cycles<br>5-Fluorouracil 200 mg/m^2/d civi x 6 months	Roth AD et al. Docetaxel, cisplatin; and fluorouracil; docetaxel and cisplatin; and epirubicin, cisplatin, and fluorouracil as systemic treatment for advanced gastric carcinoma: a randomized phase II trial of the Swiss Group for Clinical Cancer Research. J Clin Oncol 2007; 25:3217
**Nasopharyngeal CA**<br><br>**and Oral Cavity**	Docetaxel (Taxotere) 75 mg/m^2 iv over 1 hour d1<br>Cisplatin (CDDP) 100 mg/m^2 iv over 30 min-3 hours d1<br>5-Fluorouracil 1000 mg/m^2/d civi d1-4<br>Q3w x 3 cycles<br>3-8 weeks later:<br>Carboplatin (Paraplatin) AUC 1.5 iv over 1 hour qw x 7 weeks during RT<br>Concurrent radiotherapy 2 Gy/d to a total of 70-74 Gy<br>6-12 weeks later:<br>Surgical resection as needed	Posner MR et al. Cisplatin and fluorouracil alone or with docetaxel in head and neck cancer. N Eng J Med 2007; 357:1705
**Ovary CA**	Carboplatin (Paraplatin) AUC 5-7.5 iv over 1 hr d1<br>Paclitaxel (Taxol) 175 mg/m^2 iv over 3 hrs d1<br>Q3w x 6 cycles	Ozols, RF et al. Phase III trial of carboplatin and paclitaxel compared with cisplatin and paclitaxel in patients with optimally resected stage III ovarian cancer: a Gynecologic Oncology Group study. J Clin Oncol 2003; 21:3194
**Lung CA**	Paclitaxel (Taxol) 175 mg/m2 iv over 3 hours day 1<br>Cisplatin (CDDP) 70 mg/m^2 iv day 1<br>Q3w x 4 cycles<br><br><br>Cisplatin (CDDP) 100 mg/m^2 iv day 1<br>Gemcitabine (Gemzar) 1000 mg/m^2 iv days 1, 8, 15Q4w	Park JO et al. Phase III trial of two versus four additional cycles in patients who are nonprogressive after two cycles of platinum-based chemotherapy in non-small-cell lung cancer. J Clin Oncol 2007; 25:5233<br>Schiller JH et al. Comparison of four chemotherapy regimens for advanced non-small cell lung cancer. N Eng J Med 2002; 346:92
**Pancreas CA**	Gemcitabine (Gemzar)<br>1000 mg/m^2 iv over 30 min qw x 3 weeks<br>Q4w x 6 cycles<br><br><br><br>Gemcitabine (Gemzar) 1000 mg/m^2 iv over 100 min d1<br>Oxaliplatin (Eloxatin) 100 mg/m^2 iv over 2 hrs d2<br>Q2w	Neuhaus P et al. CONKO-001: final results of the randomized, prospective, multicenter phase III trial of adjuvant chemotherapy with gemcitabine vs observation in patients with resected pancreatic cancer (PC). 2008 ASCO annual meeting. LBA4504<br>Louvet, C et al. Gemcitabine in combination with oxaliplatin compared with gemcitabine alone in

		locally advanced or metastatic pancreatic cancer: results of a GERCOR and GISCAD phase III trial. J Clin Oncol 2005; 23:3509
**Prostate CA**	Docetaxel (Taxotere) 75 mg/m^2 iv over 1 h q3w x 10 cycles<br>Prednisone 5 mg po bid	Berthold DR et al. Docetaxel plus prednisone or mitoxantrone plus prednisone for advanced prostate cancer: updated survival in the TAX 327 study. J Clin Oncol 2008; 26:242
**Anal canal CA**	5-Fluorouracil 1000 mg/m^2/d civi d1-4 and 29-32<br>Mitomycin 10 mg/m^2 iv bolus d1 and 29<br>Concurrent radiotherapy 1.8 Gy/d x 5/w for 5 weeks to 45 Gy<br>4-6 weeks later, perform full thickness biopsy. If biopsy positive for residual tumor, start salvage treatment:<br>5-Fluorouracil 1000 mg/m^2/d civi d1-4<br>Cisplatin (CDDP) 100 mg/m^2 iv over 4-6 hrs d2<br>Concurrent radiotherapy 1.8 Gy/d x 5 to 9 Gy<br>6 weeks later, perform another full thickness biopsy. If still positive, do abdominoperineal resection	Ajani JA et al. Fluorouracil, mitomycin, and radiotherapy vs fluorouracil, cisplatin, and radiotherapy for carcinoma of the anal canal. JAMA 2008; 299:1914
**Germ cell tumor**	Paclitaxel (Taxol) 250 mg/m^2 iv over 24 hrs d1<br>Ifosfamide 1.5 g/m^2/d iv over 1 h d2-5<br>Cisplatin (CDDP) 25 mg/m^2/d iv over 30 min d2-5<br>Mesna 500 mg/m^2 iv before ifosfamide, and at 4 and 8 hrs after ifosfamide daily, d2-5<br>Filgrastim (Neupogen) 5 mcg/kg sc qd d7-18<br>Q3w x 4 cycles	Kondagunta, GV et al. Combination of paclitaxel, ifosfamide, and cisplatin is an effective second-line therapy for patients with relapsed testicular germ cell tumors. J Clin Oncol 2005; 23:6549
**Uterus Sarcoma**	Gemcitabine (Gemzar) 675-900 mg/m^2 iv over 90 min d1, 8<br>Docetaxel (Taxotere) 100 mg/m^2 iv over 1 h d8<br>Filgrastim (Neupogen) 5 mcg/kg sc qd d9-15<br>Q3w	Maki RG et al. Randomized phase II study of gemcitabine and docetaxel compared with gemcitabine alone in patients with metastatic soft tissue sarcoma. J Clin Oncol 2007; 25:2755
**Urinary bladder CA**	Gemcitabine (Gemzar) 1000 mg/m^2 iv over 30-60 min d1, 8 and 15<br>Cisplatin (CDDP) 70 mg/m^2 iv d2<br>Q4w x 6 cycles	von der Maase H et al. Gemcitabine and cisplatin versus methotrexate, vinblastine, doxorubicin and cisplatin in advanced or metastatic bladder cancer: results of a large, randomized, multinational, multicenter, phase III study. J Clin Oncol 2000; 18:3068
**Glioblastoma CA**	Radiotherapy to 60 Gy<br>Concurrent temozolomide (Temodar) 75 mg/m^2 po qd<br>4 weeks after radiation, continue temozolomide 150-200 mg/m2 po qd x 5 days every month	Stupp, R et al. Radiotherapy plus concomitant and adjuvant temozolomide for glioblastoma. N Engl J Med 2005; 352:987

**Ewing Sarcoma**	Cyclophosphamide (Cytoxan) 1200 mg/m^2 iv d1, followed by mesna<br>Doxorubicin (Adriamycin) 75 mg/m^2 iv bolus d1, change to dactinomycin 1.25 mg/m^2 iv d1 when total doxorubicin reaches 375 mg/m2<br>Vincristine 2 mg iv d1<br>Alternating with<br>Ifosfamide 1.8 g/m2/d iv d1-5, given with mesna<br>Etoposide (VP-16) 100 mg/m^2/d iv d1-5<br>Q3w x 17 cycles	Holcombe E et al. Addition of ifosfamide and etoposide to standard chemotherapy for Ewing's sarcoma and primitive neuroectodermal tumor of bone. N Engl J Med 2003; 348:694

Supplement Table 2. The specific characteristics of the analyzed microsatellite markers.

Name (locus)	Primer sequence (5’ to 3’)	Unit of repeats	PCR-Tm[c]	Dye	Size (bp)
TP53-alu	F...GCA CTT TCC TCA ACT CTA CA<br>R...AAC AGC TCC TTT AAT GGC AG	5	55°C	FAM	382-417
Mfd15 (D17S250)	F....GGA AGA ATC AAA TAG ACA AT<br>R...GCT GGC CAT ATA TAT ATT TAA ACC	2	50°C	VIC	147-163
Mfd41 (D17S261)	F...CAG GTT CTG TCA TAG GAC TA<br>R...TTC TGG AAA CCT ACT CCT GA	2	55°C	NED	153-172
APC (D5S346)	F...ACT CAC TCT AGT GAT AAA TCG<br>R...AGC AGA TAA GAC AGT ATT ACT AGT T	2	55°C	FAM	107-131
Bat-25	F...TCG CCT CCA AGA ATG TAA GT<br>R...TCT GCA TTT TAA CTA TGG CTC	1	55°C	NED	119-124
TP53.PCR15	F...AGG GAT ACT ATT CAG CCC GAG GTG<br>R...ACT GCC ACT CCT TGC CCC ATT C	2	62°C	PET	98-109
AFM093xh3 (D2S123)	F...AAA CAG GAT GCC TGC CTT TA<br>R...GGA CTT TCC ACC TAT GGG AC	2	58°C	PET	209-232
Bat-40	F...ATT AAC TTC CTA CAC CAC AAC<br>R...GTA GAG CAA GAC CAC CTT G	1	55°C	VIC	118-131
Bat-26	F…TGA CTA CTT TTG ACT TCA GCC<br>R…AAC CAT TCA ACA TTT TTA ACC C	1	55°C	FAM	112-127
Mfd28	F…AAC ACT AGT GAC ATT ATT TTC<br>R...AGC TAG GCC TGA AGG CTT CT	2	55°C	FAM	139-156
