# Supplementary figures and images for: Chemotherapy induced microsatellite instability and loss of heterozygosity in chromosomes 2, 5, 10, and 17 in solid tumor patients
Source: Cancer Cell Int. 2014 Nov 30;14:118. doi: 10.1186/s12935-014-0118-4 (PMC4260186; doi:10.1186/s12935-014-0118-4)

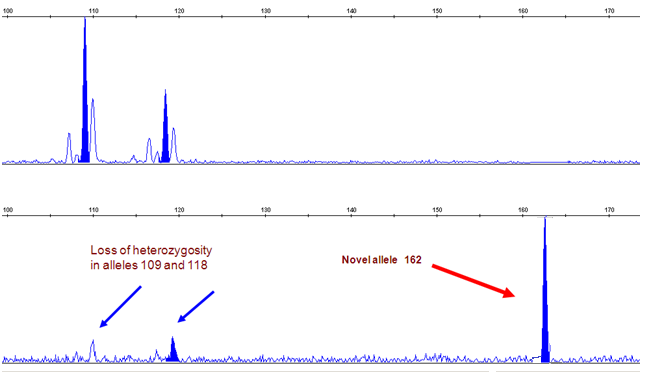

Supplement: Additional file 2: Figure S2. — Positive correlation between rates of MSI and LOH and the patient’s age. As patient’s age increases, the incidence of MSI and LOH appears to increase. [file 12935_2014_118_MOESM2_ESM.tiff]

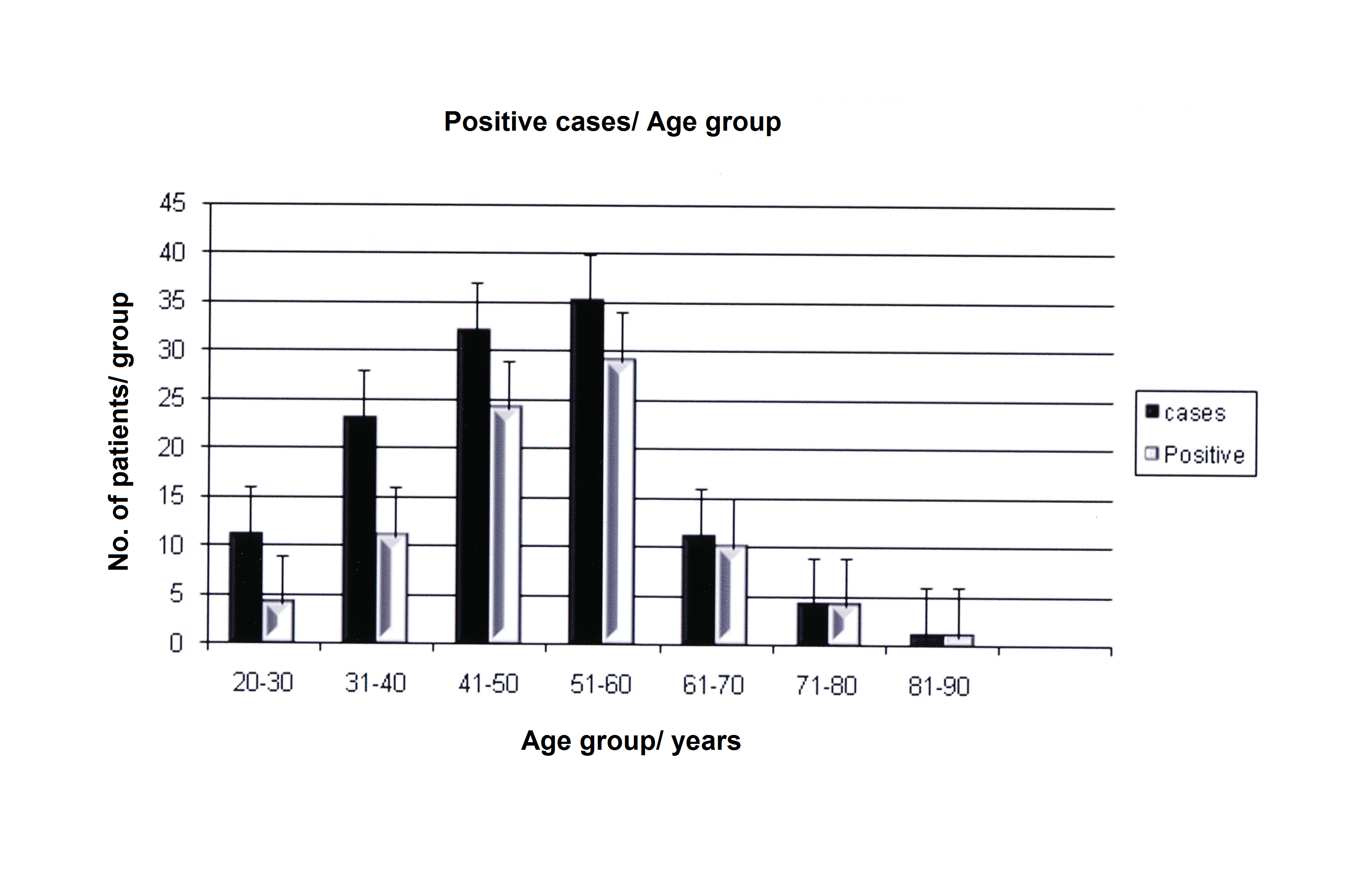

Supplement: Additional file 3: Table S1. — Frequently used chemotherapy treatments in managing the sampled solid tumors in the current study. Table S2. The specific characteristics of the analyzed microsatellite markers. [file 12935_2014_118_MOESM3_ESM.pdf]
